# Supplementary material for: MetaRibo-Seq measures translation in microbiomes
Source: Nat Commun. 2020 Jun 29;11:3268. doi: 10.1038/s41467-020-17081-z (PMC7324362; doi:10.1038/s41467-020-17081-z)
Supplement: Supplementary file 10 — Supplementary Data 7 [file 41467_2020_17081_MOESM10_ESM.zip › File2/Confidence_High_Taxonomy/377676_out.krona.html]

Javascript must be enabled to view this page.

members
magnitude
magnitudeUnassigned
count
unassigned
taxon
rank

377676\_out

72

10

SRS011302\_contig\_number\_contig-100\_41176.82291SRS011405\_contig\_number\_contig-100\_27192.27192SRS015190\_contig\_number\_contig-100\_37276.37276SRS015782\_contig\_number\_contig-100\_19267.63246SRS020328\_contig\_number\_contig-100\_32809.62576SRS023583\_contig\_number\_contig-100\_23006.23006SRS042628\_contig\_number\_contig-100\_25475.131472SRS048870\_contig\_number\_contig-100\_28561.72326SRS063190\_contig\_number\_contig-100\_16505.16506SRS140513\_contig\_number\_contig-100\_21198.21198

superkingdom
2
62

62
phylum
1239

class
186801
62

186802
order
62

31979
family
1

1485
genus
1

species
59620

SRS098571\_contig\_number\_39669
1


SRS143181\_contig\_number\_15895
species
1897048
1

family
186803
1

830
genus
1

1
1262761
species

SRS075878\_contig\_number\_9896

59
541000
family

1
292632
genus

1
species
2053618

SRS893270\_contig\_number\_contig-100\_535.41254

56
genus
216851

1
1971605
species

SRS019693\_contig\_number\_contig-100\_830.72751

55
853
species

SRS014683\_contig\_number\_contig-100\_23649.71878SRS014855\_contig\_number\_contig-100\_6657.71830SRS015264\_contig\_number\_contig-100\_2328.120574SRS015431\_contig\_number\_contig-100\_5155.424373SRS015663\_contig\_number\_contig-100\_21920.113283SRS015854\_contig\_number\_contig-100\_1687.140876SRS016335\_contig\_number\_36125SRS017307\_contig\_number\_22695SRS018623\_contig\_number\_4922SRS019068\_contig\_number\_contig-100\_8285.521933SRS020233\_contig\_number\_contig-100\_24400.150496SRS022137\_contig\_number\_contig-100\_3297.121530SRS043841\_contig\_number\_3960SRS044535\_contig\_number\_25631SRS045826\_contig\_number\_10407SRS049995\_contig\_number\_contig-100\_8268.44309SRS050752\_contig\_number\_contig-100\_16222.101778SRS050941\_contig\_number\_22544SRS053356\_contig\_number\_contig-100\_2234.264539SRS054905\_contig\_number\_contig-100\_2706.82959SRS056273\_contig\_number\_86SRS064276\_contig\_number\_contig-100\_12647.165245SRS075078\_contig\_number\_contig-100\_2529.189882SRS075773\_contig\_number\_35744SRS077454\_contig\_number\_1881SRS097889\_contig\_number\_contig-100\_10430.164971SRS097958\_contig\_number\_contig-100\_8871.107519SRS098061\_contig\_number\_3055SRS098644\_contig\_number\_19462SRS101376\_contig\_number\_contig-100\_2863.166374SRS104084\_contig\_number\_contig-100\_1560.88843SRS1041037\_contig\_number\_14083SRS1041133\_contig\_number\_contig-100\_6283.6284SRS142890\_contig\_number\_4180SRS142980\_contig\_number\_3336SRS143342\_contig\_number\_20458SRS143372\_contig\_number\_11355SRS143466\_contig\_number\_27908SRS143523\_contig\_number\_contig-100\_1468.153724SRS143722\_contig\_number\_contig-100\_2918.92229SRS143895\_contig\_number\_42724SRS144135\_contig\_number\_contig-100\_3850.182760SRS144183\_contig\_number\_contig-100\_13238.146790SRS144297\_contig\_number\_13466SRS144506\_contig\_number\_46064SRS144714\_contig\_number\_contig-100\_5813.153632SRS146812\_contig\_number\_32675SRS146813\_contig\_number\_contig-100\_23566.72608SRS147346\_contig\_number\_contig-100\_4468.328941SRS147977\_contig\_number\_1072SRS149075\_contig\_number\_1885SRS149181\_contig\_number\_11830SRS149784\_contig\_number\_contig-100\_6271.92407SRS893369\_contig\_number\_contig-100\_3843.69529SRS971276\_contig\_number\_contig-100\_6120.106973

species
1898205

SRS078242\_contig\_number\_100SRS893373\_contig\_number\_contig-100\_2849.85242
2
